# Nomograms for predicting long‐term overall survival and cancer‐specific survival in lip squamous cell carcinoma: A population‐based study

**DOI:** 10.1002/cam4.2260

**Published:** 2019-05-21

**Authors:** Chuan-Yu Hu, Zhen-Yu Pan, Jin Yang, Xiu-Hong Chu, Jun Zhang, Xue-Jin Tao, Wei-Min Chen, Yuan-Jie Li, Jun Lyu

**Affiliations:** ^1^ Clinical Research Center The First Affiliated Hospital of Xi’an Jiaotong University Xi’an China; ^2^ Stomatology Center Tongji Hospital, Tongji Medical College, Huazhong University of Science and Technology Wuhan China; ^3^ School of Public Health Xi’an Jiaotong University Health Science Center Xi’an, Shaanxi China; ^4^ Department of Pharmacy The Affiliated Children Hospital of Xi'an Jiaotong University Xi’an, Shaanxi China; ^5^ Department of Nursing Yeda Hospital Yantai China; ^6^ Department of Orthopaedics Baoji Municipal Central Hospital Baoji China; ^7^ Department of Human Anatomy, Histology and Embryology, School of Basic Medical Sciences Xi’an Jiaotong University Health Science Center Xi’an China

**Keywords:** cancer‐specific survival, lip squamous cell carcinoma, nomogram, overall survival, SEER

## Abstract

**Background:**

The goal of this study was to establish and validate two nomograms for predicting the long‐term overall survival (OS) and cancer‐specific survival (CSS) in lip squamous cell carcinoma (LSCC).

**Methods:**

This study selected 4175 patients who were diagnosed with LSCC between 2004 and 2015 in the SEER (Surveillance, Epidemiology, and End Results) database. The patients were allocated randomly to a training cohort and validation cohort. Variables were selected using a backward stepwise method in a Cox regression model. Based on the predictive model with the identified prognostic factors, nomograms were established to predict the 3‐, 5‐, and 8‐year survival OS and CSS rates of LSCC patients. The accuracy of the nomograms was evaluated based on the consistency index (C‐index), while their prediction accuracy was evaluated using calibration plots. Decision curve analyses (DCAs) were used to evaluate the performance of our survival model.

**Results:**

The multivariate analyses demonstrated that age at diagnosis, marital status, sex, race, American Joint Committee on Cancer stage, surgery status, and radiotherapy status were risk factors for both OS and CSS. The C‐index, area under the time‐dependent receiver operating characteristic curve, and calibration plots demonstrated the good performance of the nomograms. DCAs of both nomograms further showed that they exhibited good 3‐, 5‐, and 8‐year net benefits.

**Conclusions:**

We have developed and validated LSCC prognosis nomograms for OS and CSS for the first time. These nomograms can be valuable tools for clinical practice when clinicians are helping patients to understand their survival risk for the next 3, 5, and 8 years.

## 1 INTRODUCTION

Cancer of the lips accounts for more than 25% of all oral cancers,^1^ and approximately 90%‐93% of cases are squamous cell carcinoma (SCC).^2^ According to the Surveillance, Epidemiology, and End Results (SEER) statistics fact sheet on lip cancer (https://seer.cancer.gov/statfacts/html/lip.html; accessed January 4, 2019), the number of new cases in 2015 was estimated at 70 000, and the estimated death toll was 2000. Although the mortality rate is low for SCC, such carcinomas are more likely to cause damage by local invasion or cervical lymph node metastasis.^3^

The American Joint Committee on Cancer (AJCC) staging system has been widely used to determine treatment strategies for lip squamous cell carcinoma (LSCC) patients. This system is commonly used alone to predict the prognosis for an ensemble population of patients, and it has some crucial limitations since the prognosis of LSCC patients is influenced by many other factors^4^, ^5^, ^6^ such as race, marital status, and age.^7^ Ignoring these significant prognostic factors may decrease the accuracy of survival predictions, including since the survival outcomes of patients at the same AJCC stage can be vary widely. The clinical uniqueness of LSCC means that new prognostic tools are needed to increase the accuracy of survival predictions in LSCC patients.^8^

A nomogram is a convenient diagrammatic representation of a mathematical model that combines various key variables to forecast a specific outcome.^9^ Nomograms have been widely used to help surgeons develop treatment plans and assess the prognosis of various types of cancer,^10^, ^11^, ^12^ and National Comprehensive Cancer Network guidelines have introduced nomograms that perform well.^13^ The aim of the present study was to establish a comprehensive prognostic evaluation system for LSCC and validate its prediction accuracy.

## 2 METHODS

### 2.1 Patient selection

We investigated information on patients in the latest version of the SEER (covering 18 registries), by using SEER*Stat version 8.3.5 (https://seer.cancer.gov/). We searched the ICD‐O‐3 (third edition of the International Classification of Cancer Diseases) for the histological type codes of 8070‐8078 for SCC. Cases that were not confirmed by microscopy or only in an autopsy were excluded, as were those with unknown or incomplete variables. The examined variables included marital status, AJCC stage, age, race, sex, surgery status, radiotherapy status, tumor site, vital status, and cause‐specific death. We applied the sixth edition of the AJCC staging system, and we restricted our search to between 2004 and 2015 since the system was published in 2004.

There were 6603 qualified patients identified in the SEER database, of which 4175 were available after the application of a strict screening process. For the construction and validation of the nomograms, we randomly distributed 70% of the patients to the training cohort (n = 2922) and 30% to the validation cohort (n = 1253).

The main outcomes were overall survival (OS) and cancer‐specific survival (CSS). OS was defined as the interval from an LSCC diagnosis to the last follow‐up or death, without restriction as to the cause of death, while CSS was defined as the interval from an LSCC diagnosis to death due to LSCC or a review of the death status (if a patient was alive at the last follow‐up or dead from other causes). All data from the SEER database are freely available.

### 2.2 Statistical analysis

The eight pathological and clinical characteristics of age at diagnosis, marital status, race, tumor site, sex, AJCC stage, radiotherapy status, and surgery status were used to conduct the analyses. Continuous variables were expressed as mean ± SD values if they conformed to a Gaussian distribution; otherwise they were expressed as median (25th‐75th percentile) values. Categorical variables were expressed as percentages. The method of backward stepwise selection in a Cox regression model was applied to the training cohort to select variables. Based on the predictive model of prognostic factors, two nomograms were constructed for the incidence rates of OS and CSS in LSCC patients over 3, 5, and 8 years.

### 2.3 Validation of the nomograms

The nomograms were tested by measuring calibration and differentiation curves for both the training cohort (internally) and the validation cohort (externally).^14^ The prediction accuracy of each nomogram was evaluated by the consistency index (C‐index) and the area under the time‐dependent receiver operating characteristic curve (AUC). Calibration plotting was used to evaluate the agreement between the actual outcome and the predicted probability. There were two lines in the calibration plots: one was the data line and the other was a 45‐degree reference line; the discrepancy between these two lines reflects the accuracy of a nomogram.^15^

Both discrimination and calibration were evaluated using bootstrapping with 500 resamples. Decision curve analyses (DCAs) were conducted to test the clinical value of the predictive models. All of the statistical analyses were performed using SPSS software (version 24.0, SPSS, Chicago, IL, USA) and R software. A two‐sided probability value of *P* ≤ 0.05 was deemed to indicate statistical significance.

## 3 RESULTS

### 3.1 Patient characteristics

The 4175 LSCC patients screened from the SEER database were divided using the popular random split‐sample method (with a split ratio of 7:3) into 2922 in the training cohort and 1253 in the validation cohort. The median age at the time of diagnosis was 69 years in the training cohort and 67 years in the validation cohort. Most of the patients were married (73.4%), male (76.1%), white (96.0%), and at AJCC stage I (78.8%). The two cohorts comprised 81.8% of patients with tumors located on the lower lip, 10.1% with tumors on the upper lip, and 8.1% with tumors in the oral commissure. Most patients (n = 3881, 93.0%) received surgery while 3712 (88.9%) refused radiotherapy. The median follow‐up times were 50 and 51 months in the training and validation cohorts, respectively. The demographics and tumor characteristics of the patients are summarized in Table 1.

**Table 1:** Patient characteristics in the study

Variable	Training cohort	Validation cohort
Age at diagnosis	69 (53‐74)	67 (54‐75)
Race (%)		
White	2800 (95.8)	1209 (96.5)
Black	35 (1.2)	9 (0.7)
Others	87 (3.0)	35 (2.8)
Sex n (%)		
Male gender	2202 (75.4)	974 (77.7)
Female gender	720 (24.6)	279 (22.3)
Marital status n (%)		
Married	2138 (73.2)	928 (74.1)
Unmarried	400 (13.7)	164 (13.1)
Others	384 (13.1)	161 (12.8)
AJCC n (%)		
I	2307 (78.9)	984 (78.5)
II	366 (12.5)	154 (12.3)
III	128 (4.4)	61 (4.9)
IV	121 (4.2)	54 (4.3)
Tumor site n (%)		
Upper lip	307 (10.5)	114 (9.1)
Lower lip	2388 (81.7)	1029 (82.1)
Other	227 (7.8)	110 (8.8)
Surgery n (%)		
Yes	2720 (93.1)	1161 (92.7)
No	202 (6.9)	92 (7.3)
Radiotherapy		
Yes	326 (11.2)	137 (10.9)
No	2596 (88.8)	1116 (89.1)

Abbreviation: AJCC, American Joint Committee on Cancer.

### 3.2 Variable screening

After performing a univariate Cox regression analysis, data on the variables of marital status, age, sex, race, AJCC stage, surgery status, and radiotherapy status were entered into multivariate Cox regression analyses of OS. These multivariate analyses demonstrated that the age at diagnosis (hazard ratio [HR]=1.068, *P* < 0.001), being female (HR = 0.800 vs male, *P* = 0.005), being black (HR = 1.983 vs white, *P* = 0.012), AJCC stage II (HR = 1.294 vs AJCC stage I, *P* = 0.012), AJCC stage III (HR = 2.316 vs AJCC stage I, *P* < 0.001), AJCC stage IV (HR = 4.367 vs AJCC stage I, *P* < 0.001), not receiving surgery (HR = 1.515 vs surgery, *P* = 0.003), and not receiving radiotherapy (HR = 1.573 vs radiotherapy, *P* = 0.062) were risk factors for OS (Table 2).

**Table 2:** Selected variables by multivariate Cox regression analysis (training cohort) OS

Variables	Multivariate analysis		
	HR	95% CI	*P*‐value
Age at diagnosis	1.068	1.062‐1.074	＜0.001
Sex			
Male		Reference	
Female	0.800	0.684‐0.935	0.005
Race			
White		Reference	
Black	1.983	1.159‐3.393	0.012
Others	0.573	0.323‐1.016	0.056
AJCC			
	
I		Reference	
II	1.294	1.054‐1.589	0.014
III	2.316	1.750‐3.065	＜0.001
IV	4.367	3.317‐5.751	＜0.001
Surgery			
Yes		Reference	
No	1.515	1.154‐1.988	0.003
Radiotherapy			
Yes		Reference	
No	1.257	0.987‐1.597	0.062

Abbreviations: AJCC, American Joint Committee on Cancer; CI, confidence interval; HR, hazard ratio; OS, overall survival.

The multivariate analyses of CSS demonstrated that the age at diagnosis (HR = 1.030, *P* < 0.001), being black (HR = 2.413 vs white, *P* = 0.043), being unmarried (HR = 0.542 vs married, *P* = 0.035), AJCC stage II (HR = 2.182 vs AJCC stage I, *P* = 0.002), AJCC stage III (HR = 6.745 vs AJCC stage I, *P* < 0.001), AJCC stage IV (HR = 15.981 vs AJCC stage I, *P* < 0.001), and not receiving surgery (HR = 2.128 vs surgery, *P* < 0.001) were risk factors for CSS (Table 3).

**Table 3:** Selected variables by multivariate Cox regression analysis (training cohort) CSS

Variables	Multivariate analysis		
	HR	95% CI	*P*‐value
Age at diagnosis	1.030	1.017‐1.043	＜0.001
Race			
White		Reference	
Black	2.413	1.027‐5.668	0.043
Others	1.117	0.454‐2.749	0.810
Marital status			
Married		Reference	
Unmarried	0.542	0.306‐0.958	0.035
Others	1.181	0.708‐1.970	0.525
AJCC			
I		Reference	
II	2.182	1.338‐3.560	0.002
III	6.745	4.016‐11.323	＜0.001
IV	15.981	10.326‐24.733	＜0.001
Surgery			
Yes		Reference	
No	2.128	1.337‐3.384	0.001

Abbreviations: AJCC, American Joint Committee on Cancer; CI, confidence interval; CSS, cancer‐specific survival; HR, hazard ratio.

### 3.3 Prognostic nomograms for OS and CSS

**Figure 1:**
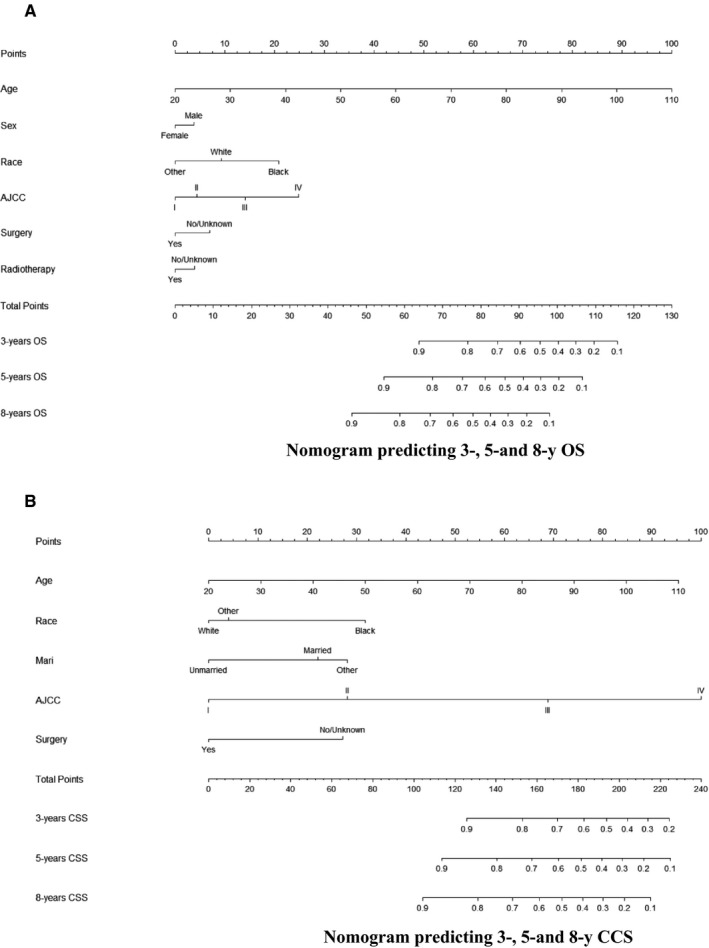
A and B, Nomogram predicting 3‐, 5‐, and 8‐y OS and CSS. CSS, cancer‐specific survival; Mari, marital status; OS, overall survival

Based on selected variables with HRs, a nomogram was established that contains all of the important independent factors for predicting the 3‐, 5‐, and 8‐year OS rates in the training cohort. The OS nomogram indicated that the age was the strongest factor influencing the prognosis, followed by the AJCC stage, race, surgery status, sex, and radiotherapy status (Figure 1A). The nomogram for predicting the 3‐, 5‐, and 8‐year CSS rates was developed using the same method (Figure 1B). In the CSS nomogram, the AJCC stage was the strongest influencing factor, followed by age, race, surgery, and marital status.

Each variable is given a score on the points scale of each nomogram. The scores for all of the variables are added to obtain the total score, and a vertical line is dropped down from the total‐points row to estimate the probability of surviving for 3, 5, and 8 years.

### 3.4 Performance of the nomograms

**Figure 2:**
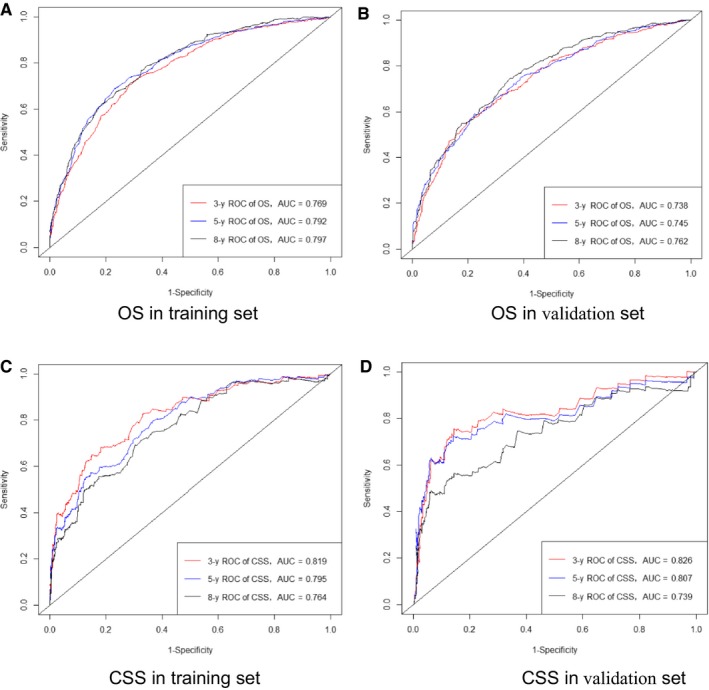
ROC curves. The ability of the model to be measured by the AUC. A, Came from the training set of OS, and (B) came from the validation set of OS; C, came from the training set of CSS, and (D) came from the validation set of CSS. CSS, cancer‐specific survival; OS, overall survival

**Figure A1:**
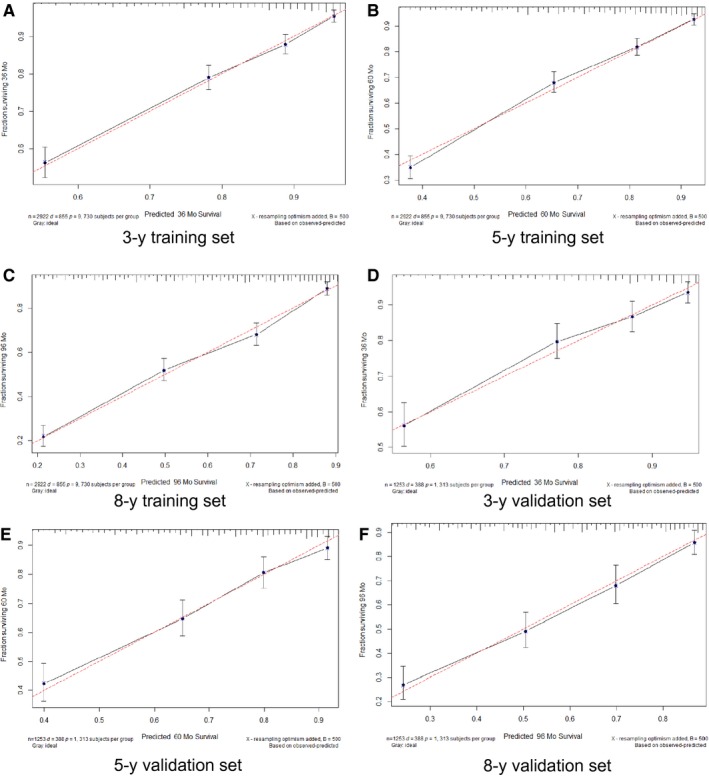
OS calibration plots. Show the relationship between the predicted probabilities base on the nomogram and actual values of the train set (A‐C) and validation set (D‐F). OS, overall survival

**Figure A2:**
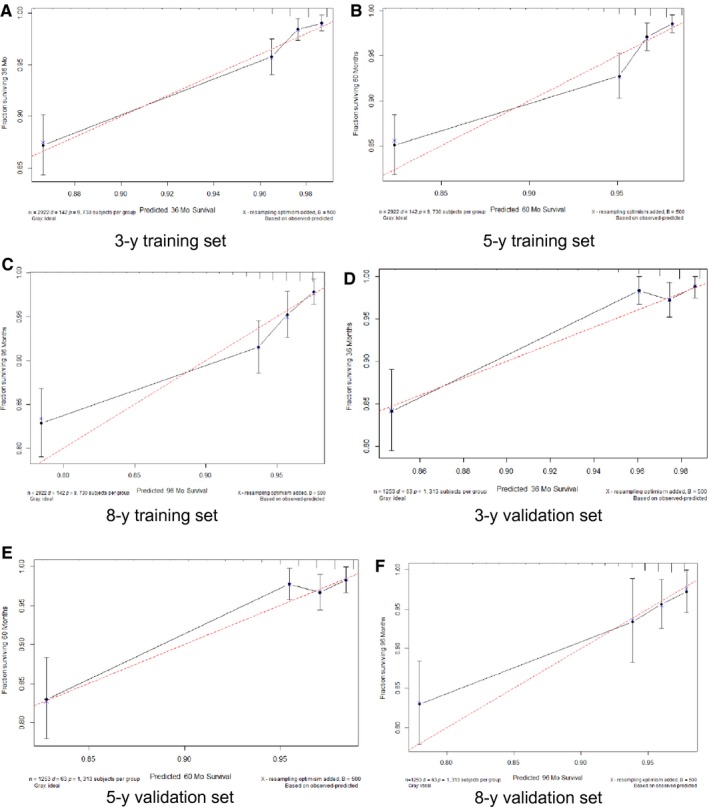
CSS Calibration plots. Show the relationship between the predicted probabilities base on the nomogram and actual values of the train set (A‐C) and validation set (D‐F). CSS, cancer‐specific survival

The C‐index for the OS nomogram was 0.748 in the training cohort and 0.723 in the validation cohort. The AUC values for the training cohort (0.769, 0.792, and 0.797 for the 3‐, 5‐, and 8‐year OS, respectively) and validation cohort (0.738, 0.745, and 0.762) indicated the good discriminative ability of the model (Figure 2A,B). The C‐index for the CSS nomogram was 0.783 in the training group and 0.799 in the validation cohort. The AUC values for the training cohort (0.819, 0.795, and 0.764 for the 3‐, 5‐, and 8‐year CCS, respectively) and validation cohort (0.826, 0.807, and 0.739) indicated the good discriminative ability of the model also for the CSS nomogram (Figure 2C,D). Calibration plots of the OS and CSS nomograms showed that the predicted 3‐, 5‐, and 8‐year survival probabilities for the training and validation cohorts were almost identical to the actual observations (Figures A1 and A2).

### 3.5 Decision curve analysis

**Figure A3:**
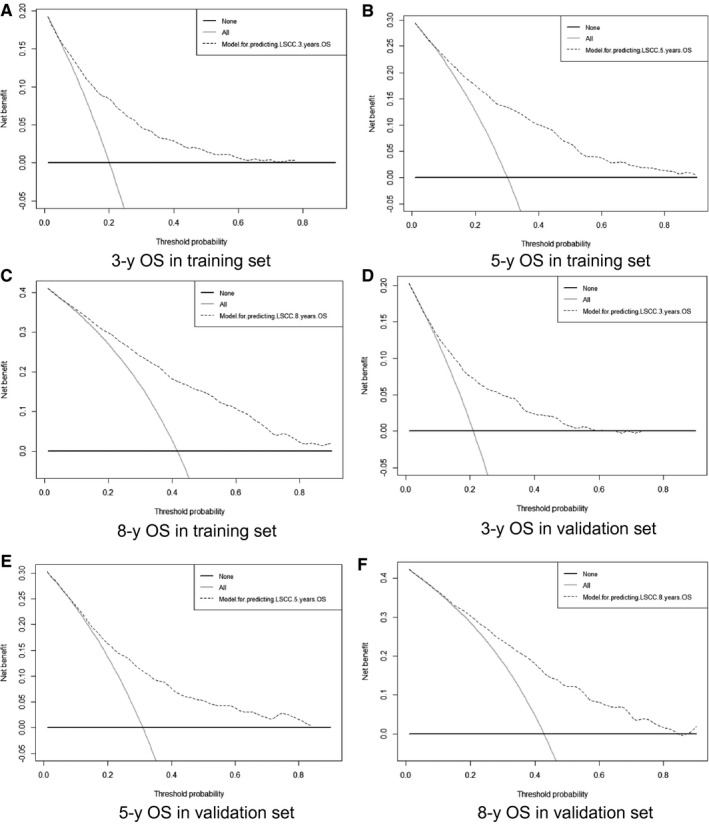
Decision curve analysis (DCA) of overall survival (OS) In the figure, the abscissa is the threshold probability, the ordinate is the net benefit rate. The horizontal one indicates that all samples are negative and all are not treated, with a net benefit of zero. The oblique one indicates that all samples are positive. The net benefit is a backslash with a negative slope. A‐C, Came from the training set, and (D‐F) came from the validation set.

**Figure A4:**
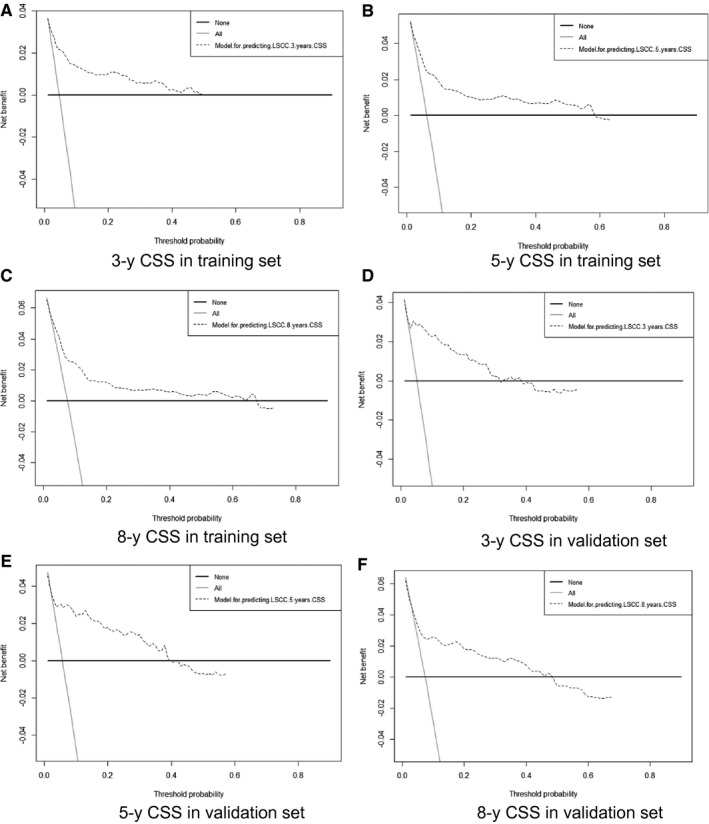
Decision curve analysis (DCA) of cancer‐specific survival (CSS) In the figure, the abscissa is the threshold probability, the ordinate is the net benefit rate. The horizontal one indicates that all samples are negative and all are not treated, with a net benefit of zero. The oblique one indicates that all samples are positive. The net benefit is a backslash with a negative slope. A‐C, Came from the training set, and (D‐F) came from the validation set.

The 3‐, 5‐, and 8‐year DCA curves indicated that both models yielded net benefits in the training and validation cohorts (Figures A3 and A4).

## 4 DISCUSSION

LSCC is a common malignant tumor of the head and neck that accounts for about 30% of oral cancers.^2^ The rate of cervical metastasis from SCC of the lip reportedly ranges from 4% to 15%.^16^, ^17^, ^18^ However, there is insufficient information for predicting the OS and CSS in patients with LSCC.^19^ Although the AJCC staging system has significant predictive power for the prognosis of LSCC patients, it does not include some important risk factors such as sex, marital status, age, race, surgery status, and radiotherapy status. In contrast to the AJCC staging system being based on assessments of the prognosis in risk groups, we sought to develop and validate predictive nomograms for predicting the 3‐, 5‐, and 8‐year OS and CSS rates in individual LSCC patients. By including readily available and important prognostic factors, such nomograms can provide quantifiable prognostic predictions for each patient.^20^

The proposed nomograms are easy‐to‐use clinical tools that will facilitate the promotion of patient counseling and personalized treatments. As an example, consider the following two LSCC patients at AJCC stage III: patient 1 is a 69‐year‐old black married male who received surgery only, while patient 2 is a 58‐year‐old white unmarried female who underwent both surgery and radiation. Applying the AJCC staging manual would produce the identical prognosis for both of these patients.^21^ However, the results obtained by applying our new nomograms are different: the 3‐, 5‐, and 8‐year predicted OS rates were 45%, 24%, and 10%, respectively, for patient 1, and 85%, 77%, and 63% for patient 2; the corresponding CSS rates were 66%, 53%, 48%, 94%, 90%, and 86%, respectively. Furthermore, the eighth version of the AJCC staging system indicates that prognoses would be evaluated while also considering nomograms in future versions.^22^

The nomograms proposed in the present study contain several independent prognostic factors that are conventionally used in clinical practice. Age has the highest score in the both nomograms. Multivariable analyses have found older age to be an independent risk variable for both OS and CSS, clearly indicating that older patients have a lower survival rate.^23^, ^24^, ^25^ Also, a higher AJCC stage has been associated with greater harm to patient survival.^21^ Our study found the same tendencies, with the nomogram score increasing with the AJCC stage. However, our two nomograms indicated that the AJCC stage is the second most important factor for OS but the first most important one for CSS. This finding is reasonable given that CSS is focused specifically on the cancer itself.

We found that the OS was higher for females than for males (Table 2), whereas there was no statistically significant sex‐related difference in CSS (Table 3). This outcome resembles previous findings.^26^ Males are more likely to drink alcohol and smoke, which are closely associated with not‐cancer‐specific death. LSCC most commonly affects white individuals,^19^ accounting for 96% of all cases in the present study, but most of them had a good prognosis.

Studies of African Americans have found that the overall incidence of SCC is lower in the head and neck, while the incidence of more aggressive and advanced mutations is higher, which may contribute to a higher HR.^27^, ^28^ This is also in consistent with our results. Surgery is generally recommended as a first‐line treatment for lip carcinoma,^29^ and surgical resection with negative margins is the mainstay remedy for LSCC,^30^ and both the OS and CSS nomograms show the same importance of surgery as an individual predictive factor.

We have developed easy‐to‐use nomograms for predicting OS and CSS in LSCC patients at 3, 5, and 8 years. Our nomogram model contains risk factors that are readily available and collected through historical records. The clinical applicability and ease of use are among the most important advantages of the nomograms that we have constructed.

We validated the accuracy of our new nomograms using the C‐index and calibration curves in both a modeling cohort (internally) and validation cohort (externally). The C‐indexes for the 3‐, 5‐, and 8‐year OS and CSS rates were 0.748 and 0.783, respectively, in the internal validation, and 0.723 and 0.799 in the external validation. All of these C‐indexes exceed 0.7, and there was excellent coherence between the calibration curves and the 45‐degree ideal lines (Figure 2).^15^ The plots resembling 45‐degree lines indicate that the nomogram predictions were well calibrated both in the training and verification cohorts (Figures A1 and A2).

Some studies have demonstrated the benefits of the latest method of DCA and recommend its use.^31^, ^32^ The results of our study showed that the 3‐, 5‐, and 8‐year DCA curves for both OS and CSS indicate the net benefits with a good performance in both the training and validation cohorts.

### 4.1 Limitations

The present study based on a large population had some strength, but certain limitations also need to be addressed. First, there was no information about the use of different surgical procedures (neck dissection or other) in the SEER database, or certain important clinical pathological parameters related to prognosis including vascular invasion and the surgical margin.^14^ Second, information about some prognostic factors such as chemotherapy and tumor markers such as EGFR, HPV, and P53^33^, ^34^, ^35^ is also not available. Third, the nomograms were established using retrospective data obtained from the SEER database, which is associated with the potential hazard of selection bias. Finally, the predicted values calculated using the nomogram are for reference use by clinicians only, rather than representing absolutely accurate prognoses. We plan to perform future prospective studies to test the nomograms with the aim of compensating for these limitations.

## 5 CONCLUSIONS

Using a large population‐based cohort, we have established and validated two nomograms for estimating the 3‐, 5‐, and 8‐year OS and CSS rates in patients with LSCC for the first time. These nomograms were found to be accurate and show strong predictive power in both internal and external validation tests. They are precise and easy to implement, and hence can provide clinicians with reference information for determining customized clinical treatment options and providing personalized prognoses.

## CONFLICT OF INTEREST

The author reports no conflict of interest in this work.

## Data Availability

I confirm that my article contains a Data Availability Statement even if no data are available (list of sample statements) unless my article type does not require one. I confirm that I have included a citation for available data in my references section, unless my article type is exempt.
